# Comparative analysis of tRNAs across mouse strains reveals gene-specific conservation, divergence, and copy number variation

**DOI:** 10.1186/s12864-026-12787-8

**Published:** 2026-03-26

**Authors:** Andrew D. Holmes, Upasna Sharma

**Affiliations:** 1 Department of Molecular, Cell and Developmental Biology, University of California, Santa Cruz, CA 95064 USA; 2 RNA Center, University of California, Santa Cruz, CA 95064 USA; 3 Genomics Institute, University of California, Santa Cruz, CA 95064 USA

**Keywords:** TRNA genes, Evolution, Genomics, Mouse strains

## Abstract

**Background:**

Transfer RNAs (tRNAs) are essential for translation in all forms of life. tRNA genes in eukaryotes exist in multiple identical and non-identical copies across the genome to contribute to the tRNA repertoire of a cell. Given their central role in translation, tRNA genes are often assumed to be highly conserved across mammalian genomes. However, the true extent of tRNA gene conservation, divergence, and copy number variation has not been systematically explored. This study examines the evolution of tRNAs and tRNA-like elements (SINE elements, pseudo-tRNAs and other sequences similar to tRNAs) across closely related mouse strain genomes to understand how these loci diverge over short evolutionary timescales. By integrating previously published RNA Polymerase III Chromatin Immunoprecipitation followed by Sequencing (ChIP-Seq) data with a systematic identification of orthologous tRNA genes, we assess how actively transcribed tRNAs and tRNA-like elements vary between strains. We also compare the tRNA gene complement between strains to determine how the tRNA copy number changes.

**Results:**

We find that even actively transcribed tRNA genes can differ between closely related mouse strains, sometimes in ways that render them non-functional. While certain tRNAs exhibit rapid evolution and extensive sequence variation, others remain completely conserved. We also identify a subset of single-copy tRNAs that are remarkably conserved not only across mouse strains but even across mammals, suggesting specialized functions. Finally, our analysis infers large-scale gene conversion events among tRNAs and tRNA-like elements between mouse strains, offering new insight into the mechanisms shaping tRNA gene conservation and diversification.

**Conclusions:**

Our findings demonstrate that tRNA genes can be both gained and lost even among closely related mouse strains, revealing a dynamic and complex landscape of tRNA evolution. We observe a mixture of rapidly evolving and highly conserved tRNAs, reflecting distinct evolutionary pressures. Our results also show some evidence for the concerted evolution hypothesis, where multicopy tRNAs that can mutate are kept in sync through gene conversion. These findings not only illuminate the dynamics of tRNA gene evolution within a species but also provide a foundation for future efforts to experimentally modify tRNA loci in the genome by highlighting which tRNA genes are most likely to be functionally or evolutionarily significant.

**Supplementary Information:**

The online version contains supplementary material available at 10.1186/s12864-026-12787-8.

## Background

Transfer RNAs (tRNAs) are essential components of the translation machinery, linking codons to amino acids and thereby ensuring accurate and efficient protein synthesis. Beyond their canonical role in translation, tRNAs and their cleavage product, known as tRNA fragments or tRNA-derived RNA (tDRs) [1], are increasingly recognized as regulators of gene expression, stress response, developmental processes, and intergenerational epigenetic inheritance [2, 3]. tRNA genes form one of the largest gene families, with several hundred predicted functional members in most genomes. Since only 64 codons must be decoded, tRNA genes in eukaryotes exist in multiple identical and non-identical copies, with many tRNAs sharing the same anticodon or belonging to the same isoacceptor group, to allow for faster transcription and functional redundancy. Given their central role in cellular physiology, tRNA genes are often assumed to be highly conserved across mammalian genomes [4–6]; however, studies in human populations have revealed unexpected tRNA diversity, including sequence and copy number variations [7–9]. Despite these key insights, the true extent of tRNA gene conservation, divergence, and copy number variation across closely related species or strains has not been systematically explored.

A major challenge in analyzing tRNA gene family evolution lies in accurately defining homology relationships among loci across species. Because subsets of functionally equivalent tRNAs often have the same sequence, sequence-based approaches alone are incapable of distinguishing true orthologs from paralogs [10]. As a result, analyses of tRNA evolution require additional contextual information, such as structural information, genomic position, and transcriptional signature.

The structure of cytosolic tRNA is conserved in all domains of life. tRNAs generally form a “cloverleaf” secondary structure, with the acceptor arm, the D arm, the anticodon arm, the T arm, and, in some tRNAs, a variable loop [11, 12]. This well-defined, conserved structure of tRNAs has allowed numbering all specific bases using the “Sprinzl” numbering system, which can then be used across tRNAs to identify specific bases that are modified, recognized by tRNA-aminoacylases, or differ in sequences [13]. Moreover, tRNA genes have generally been predicted in assembled genomes based on their sequence and structure being similar to known tRNAs, as established with covariance models [14]. These tools predict both bona fide tRNA genes and tRNA-like elements by scoring their sequence and secondary structure similarity to canonical tRNA models using a covariance model. This score thus allows for tRNA genes to be ranked by their best-fit and can be used to differentiate tRNA-like elements (low scoring) from transcribed and active tRNAs (high scoring). The tRNA-like elements can include pseudo-tRNAs, truncated tRNAs, mitochondrial tRNAs copied into the nuclear genome, regions that randomly match tRNAs, and tRNA-like SINE elements [15]. Notably, while tRNA-like elements are mostly inactive or non-functional, a subset of SINE elements show active chromatic marks in chicken and zebra finch genomes, suggesting a potential to be transcribed to contribute to the tRNA pool [16]. Covariance models have also been used to identify tRNAs by amino acids, which, even outside the anticodon sequence, have distinct profiles for each amino acid [17]. This approach also has some limitations, as even identical tRNA genes can differ at the level of transcription.

tRNA genes are transcribed by RNA polymerase III (Pol III) using internal promoters with conserved A box and B box sequences [18]. However, these sequence features are not sufficient to dictate tRNA expression, as identical tRNA copies can be active or inactive [19]. While the full determinants of tRNA gene activity are not known, the local genomic context, such as chromatin state, presence in tRNA arrays and CpG density, in combination with internal sequences, provides specificity [20]. One way to identify transcribed tRNAs is by measuring Pol III binding at tRNA genes, wherein Pol III occupancy can be used as a proxy for tRNA transcription [19, 21–25]. Moreover, tRNA expression is globally regulated by specific regulatory factors, such as Maf1, a repressor of Pol III [26]. Furthermore, tRNAs can be expressed in a tissue-specific manner, although the factors contributing to this specificity remain unclear. For example, tRNA-Arg-TCT-4 is highly expressed in the brain, as observed by Pol III binding and transcript abundance across tissues [27, 28]. Together, these observations underscore the need for integrative analyses that combine sequence, structural, and transcriptional information to accurately define functional tRNA loci and explore how evolutionary forces shape their diversity and regulation across genomes.

Notably, tRNAs exist in many copies in any given eukaryotic genome, allowing for greater transcript redundancy, amplifying transcription, and possibly translation-independent functions. These tRNA sequences show conservation both between orthologs in different species and within, with genomes containing multiple identical copies of certain tRNAs and a single copy of others [29, 30]. While the tRNA genes themselves are highly conserved and often present in multiple copies within genomes, the flanking sequences incorporated into pre-tRNA transcripts show substantial divergence across orthologs and paralogs [20]. Importantly, tRNAs are also known to vary in copy number among individual humans, despite being highly conserved [9]. It has been suggested that the maintenance of multiple identical tRNA gene copies across the genome, beyond what may be necessary for function, could result from a process of concerted evolution. According to this idea, which has also been suggested for ribosomal RNA genes [31], the tRNA genes spread across the genome are kept identical in sequence through gene conversion. While this has been induced in yeast tRNAs [32], little is known about this process.

Previous studies of tRNA gene deletions in mice have shown that while some tRNAs can compensate for the loss of others, certain deletions cause non-viable or incompletely rescued phenotypes even when identical copies exist elsewhere in the genome [33]. These observations suggest that tRNA genes are under selective pressure to preserve both their genomic complement and locus-specific functions, and that even sequence-identical tRNAs can differ in biological function. Yet, the circumstances under which individual tRNA genes become uniquely essential remain poorly understood. To explore these questions, here we analyze predicted tRNA genes and tRNA-like elements across 17 mouse strain genomes, which provide a suitable model for studying evolution on a relatively short timescale. By integrating covariance scoring with previously published Pol III ChIP-seq data, we identified actively transcribed tRNA genes in the reference genome and uncovered their sequence and copy-number variation across 17 strains. We show that while the majority of tRNAs are conserved, subsets of tRNAs exhibit copy number differences, sequence divergence, and even strain-specific gain or loss. Moreover, we identify a set of single-copy active tRNAs that are deeply conserved across multiple strains and mammals, implicating specialized critical functions. Finally, our results also show some evidence for the concerted evolution of tRNA genes. Together, these findings provide new insights into the evolutionary dynamics of the mammalian tRNA repertoire and establish a foundation for linking tRNA variation to functional consequences in translation and beyond.

## Methods

### Generating mouse ortholog sets

Mouse tRNA annotations were gathered from the genomic tRNA database for the mm10 assembly and all other sequenced strains, including high-confidence, atypical predictions, and tRNA-like predictions [34]. Annotated genes in this full tRNA gene set were classified into two groups: “high-scoring tRNAs” (defined as sequences with scores greater than 50, without filtering for repetition, repetitiveness, or codon/body matching) and low-scoring “tRNA-like elements.” The latter category was intended as a broad class encompassing SINEs, pseudogenes, and other sequences with partial similarity to tRNAs, and was used for some downstream analyses. These annotations were compared to a mouse strain cactus graph alignment [35] to get sequences for all orthologous regions to any mouse strain tRNA. Ortholog sets were compared to ensure that all relationships were bidirectional. Multi-strain sequence alignments of all tRNAs and tRNA-like elements with 20 flanking bases were taken from a previously published cactus alignment (http://hgdownload.soe.ucsc.edu/hubs/mouseStrains/mouseStrains_1509.hal) for all tRNAs in all other genomes, removing duplicate alignments and merging regions where necessary to remove discontinuities, and removing sequences < 50 bases long. We used Cactus genome graphs and halLiftover version 2.6.7 for coordinate projection based on the cactus graph multi-genome alignments, locating ortholog sets where all genes in the full tRNA-gene set were orthologous to all others in that set. To further ensure ortholog assignment accuracy, we independently validated mappings using pairwise LASTZ v1.04.52 alignments of each tRNA together with 100 bp of flanking sequence and requiring a minimum alignment length of half the total sequence to ensure these were not merely aligning identical duplicated gene sequences but were aligning flanking sequence. This validation step excluded only 9 candidate pairs involving high-scoring mm10 tRNAs (8 sequence-identical pairs and 2 containing a single nucleotide mismatch in all tRNA with a score > 50), which represent less than 0.2% of these 5,857 total orthologous pairs retained for downstream analyses. These analyses thus indicated that the halLiftover-based approach provides a highly robust and accurate ortholog set.

Next, the set of unique sequences generated with the halLiftover-based approach was run through tRNAscan-SE 2.0.6 [17] with the minimum score set to 10, to detect the full tRNA gene set including both tRNAs and tRNA-like elements. The output of tRNAscan-SE was used to generate the projected mature tRNA sequence for each of these by removing the intron, adding the CCA tail, and adding the post-transcriptional “G” base to histidine tRNAs. Sequences with N bases were also discarded at this point to remove ambiguity. tRNAscan-SE scores were also taken at this point to evaluate the quality of the tRNA, and sequences with no tRNAscan-SE result were discarded. These sequences were then aligned using infernal cmalign 1.1.2 [14] to a mature eukaryotic tRNA sequence covariance model [17]. Sequence differences were calculated for each tRNA or tRNA-like element relative to all other high-scoring orthologous tRNAs (score > 50), as well as relative to the “high-confidence set” of high-scoring mouse reference genome tRNAs from this alignment. Orthologs not present in the set were classified as “missing” if no tRNA alignment could be found in that strain and “pseudo” if no tRNA or tRNA-like sequence could be detected by tRNAscan-SE. This full set of genes, including low-scoring genes, was used for subsequence tRNA analysis. To identify tRNA differences consistent with gene conversion, we compared tRNA copy numbers between genome pairs and focused on cases in which two orthologous loci were present in multiple copies in both genomes. tRNA pairs were removed if one sequence was a substring of the larger sequence.

### Mouse RNA polymerase III ChIP-seq analysis

Mouse RNA Pol III ChIP-seq data was generated in a previous study that examined Pol III binding loci in regenerating mouse liver by using an antibody targeting the RPC4 subunit (also known as POLR3D) [36]. Reads from this dataset were mapped to the mouse mm10 reference genome using the Bowtie2 v2.5.3 with default parameters [37]. To minimize spurious read assignments arising from the highly repetitive nature of tRNA genes, only uniquely mapping reads were retained and multi-mapping reads were discarded. For quantification of Pol III occupancy, reads overlapping annotated tRNA and tRNA-like loci extended by 50 bp both upstream and downstream were counted, allowing incorporation of flanking genomic sequence to improve locus-specific read assignment. To more easily display binding, a Pol III score was calculated as the log2-ratio of read counts plus pseudocount of 20 in the regenerating liver RPC4 pulldown to the IgG (no-antibody) control pulldown. Values below 0 were set to 0 and values above 6 were capped at 6. To assess antibody specificity and experimental robustness, we analyzed two additional Pol III ChIP-seq datasets: one using the same RPC4 antibody in liver tissue [38], and another profiling alternative Pol III subunits (POLR3G, POLR3L, and POLR3C) in stem cells [39], both counted with the same mapping and scoring system as above.

## Results

### RNA polymerase III binding analysis confirms active tRNA gene predictions

**Fig. 1:**
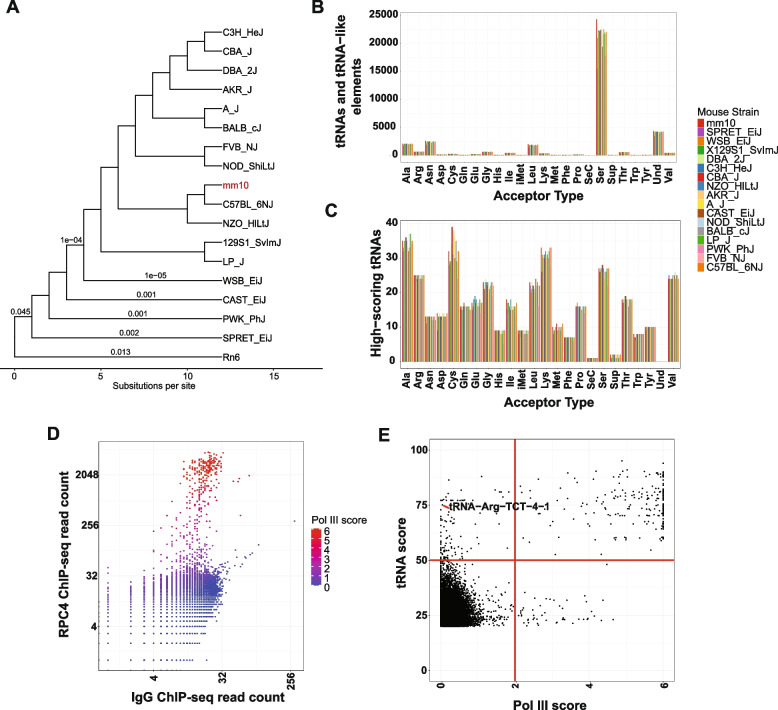
RNA Pol III occupancy-based prediction of transcribed tRNA elements: **A** Phylogenetic tree of 17 mouse strains with rat included as an outgroup. Phylogenetic tree topology and branch lengths are from the guide tree used in the cactus alignment (adapted from the tree used in the HAL alignment). Branch lengths are in substitutions per site. Short internal branches that fell below the resolution of the guide tree are not labeled. **B** Total counts of annotated tRNAs and tRNA-like elements across assembled mouse strains and rat. **C** Counts of high-scoring tRNAs by acceptor type for each strain. **D** RNA polymerase III (Pol III) ChIP-seq signal in regenerating liver 48 h after partial hepatectomy, based on previously published data. Data points are colored by Pol III score, calculated as the ratio of RPC4 ChIP-seq reads to IgG control reads. **E** Comparison of tRNA scores derived from tRNAscan-SE (y-axis) and Pol III scores derived from RPC4 ChIP-seq data (x-axis). Red lines indicate threshold values used for subsequent analyses

To study the number and conservation of tRNA genes across mouse strains, we retrieved the full tRNA-gene set, which includes both tRNAs and tRNA-like elements, from the assembled genomes of the following mouse strains: 129S1/SvImJ, A/J, AKR/J, CAST/EiJ, CBA/J, DBA/2 J, FVB/NJ, NOD/ShiLtJ, NZO/HlLtJ, PWK/PhJ, CBA/J, WSB/EiJ, SPRET/EiJ, C57BL/6NJ, BALB/cJ**,** IP/J**,** and C3H/HeJ (Fig. 1A). tRNA-like SINE elements are highly abundant in rodent genomes compared to the human genome [40] and could cause the count of tRNA-like elements to vary wildly between strains. However, our analysis revealed that the total counts of tRNAs and tRNA-like elements for a given isotype of tRNA remain broadly the same across the mouse strains, with Ser, Leu, Asn, and Ala isotypes being the most abundant across all strains (Fig. 1B).

To differentiate between active tRNA genes and inactive tRNA-like elements, we used their covariance score as calculated by tRNAscan-SE [17], which measures how close their sequence and structure are to known active tRNAs. We used a tRNA covariance score (tRNA score) cutoff of 50, to create a putative set of transcribed and functional tRNAs for the reference genome that can be easily extended to other strains. This resulted in a greatly reduced set of putative transcribed tRNAs in these genomes, reducing from 39,052 to 390 tRNAs in the mouse mm10 reference genome. (Fig. 1C). For instance, without a tRNA score cutoff, there are 24,296 Serine tRNA genes and tRNA-like elements, and this number reduces to 27 when only considering the high-scoring, potentially active tRNA genes. To evaluate whether a tRNA score threshold of 50 reliably identifies actively transcribed tRNA genes, we analyzed published RNA Pol III ChIP-seq data [36], measuring binding of the RNA Pol III subunit RPC4 (also known as POLR3D) to the reference mouse genome during liver regeneration following partial hepatectomy. During this regenerative state, most tRNA genes are expected to be transcriptionally active, providing a robust reference for Pol III occupancy. RNA Pol III was specifically enriched in 326 tRNA genes (out of 39,052 annotated tRNAs and tRNA like elements), quantified by comparing RPC4 ChIP-seq read counts to the no-antibody control (Fig. 1D). For each gene, a “Pol III score” was calculated as the Log2 ratio of RPC4-enriched reads to control reads, representing relative Pol III occupancy at that locus. Comparison of Pol III scores with tRNA scores revealed a strong positive correlation (Fig. 1E), indicating that covariance scores reliably predict actively transcribed tRNA genes in our tRNA gene set. To assess robustness of these results, we analyzed an independent Pol III ChIP-seq dataset generated using the same anti-RPC4 (POLR3D) antibody [38], which showed strong agreement with our primary dataset (*r =* 0.85) (Supplementary Figure S1A). We also compared datasets targeting additional Pol III subunits (POLR3C, POLR3G, and POLR3GL) in stem cells [39], observing high agreement for POLR3C and POLR3GL and the expected embryonic stem cells-specific enrichment for POLR3G [41] (Supplementary Figure S1 B-D). Together, these results support the reliability of the ChIP-seq dataset used to identify Pol III–bound tRNA loci.

Despite the overall concordance, a subset of loci showed discordance between tRNA and Pol III scores. Using a Pol III score cutoff of 2, we identified 116 tRNA genes with high covariance scores but low Pol III occupancy, and 51 low-scoring tRNAs that exhibited Pol III binding in regenerating liver. Because the Pol III ChIP-seq data were derived from liver tissue, only tRNAs active in this tissue can be detected. Accordingly, some high-scoring tRNAs with low Pol III scores likely reflect tissue-specific expression. For example, the brain-enriched tRNA-Arg-TCT-4 [28] lacked Pol III binding in liver at this threshold but is Pol III–bound and transcriptionally active in brain tissue [27, 28] (Fig. 1E). Nevertheless, the majority of reference genome tRNAs with covariance scores ≥ 50 exhibited Pol III binding, supporting the use of covariance scoring as a viable predictor of active tRNA genes across mouse strains.

### Single-copy conserved tRNAs across mouse strains, implicating specialized function

**Fig. 2:**
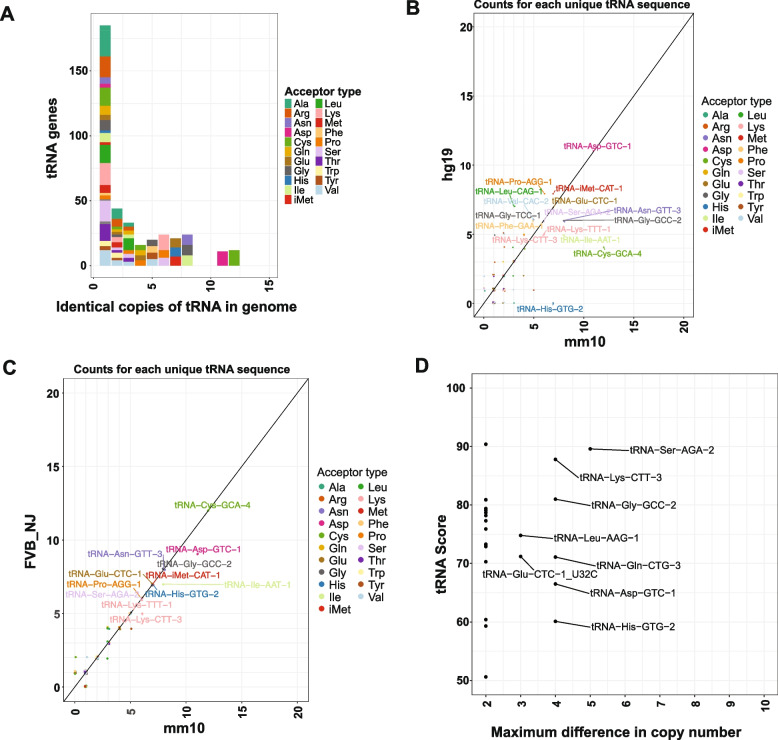
tRNA copy number variation across mouse strain assemblies: **A** Histogram showing the distribution of tRNA gene copy numbers for all high-scoring tRNAs in the mm10 reference assembly. **B** Comparison of tRNA copy numbers for high-scoring tRNAs between the mm10 and hg19 assemblies. **C** Comparison of tRNA copy numbers for high-scoring tRNAs between the mm10 reference assembly and the FVB/NJ strain assembly. **D** Relationship between tRNA score (y-axis) and the range of tRNA copy number variation across mouse strain assemblies, calculated as the difference between the maximum and minimum copy number per tRNA (excluding rat)

tRNA gene sequences can occur as multiple identical copies spread out in the genome or as single copies, also known as “orphans” [30]. To examine if tRNA copy number varied across mouse strains, we identified single-copy and multiple-copy tRNAs across the 17 mouse strains [42], removing any tRNAs with ambiguous bases (Table S1). To enable cross-genome comparisons of tRNA scores, we categorized tRNAs into high-scoring (> 50) and low-scoring groups (Table S2). We found that 52% (205) of all high-scoring mouse tRNA loci have a tRNA sequence that exists in multiple identical copies in the current reference assembly, with the remaining existing as unique single-copy tRNAs (185) (Fig. 2A). Moreover, consistent with tRNAs being slow-evolving [4], these multicopy tRNA sequences also exist in multiple copies in the human genome (Fig. 2B), although the exact number of copies varies for some. As expected, a comparison of different mouse strains to the reference genome revealed that tRNA copy numbers remained mostly constant between mouse strains. For instance, the dominant multicopy tRNA genes like tRNA-Cys-CGA-4 and tRNA-Gly-GCC-2 exist in identical copy numbers in the reference mm10 genome and FVB/NJ strain genome (Fig. 2C). However, we also found that tRNAs can vary in copy number across strains. For example, tRNA-Asp-GTC-1 is present in 11 copies in mm10 and 9 copies in FVB/NJ. Across all strain comparisons, we found extreme cases where tRNA copy number can differ by up to 5 copies between any two mouse strains, as in the case of tRNA-Ser-AGA-2 (Fig. 2D). Due to the suboptimal genome assemblies of some of these strains and the repetitive nature of tRNAs, some tRNA copies existing in unassembled regions may be missed in the analysis. However, we did not observe highly variable tRNA copy numbers (most tRNAs show the same copy numbers across strains) or changes in the dominant tRNA decoders across strains, as in the case of tRNA-Cys-CGA-4. Thus, our analysis indicates that these assemblies are accurately distinguishing single-copy and multicopy tRNAs, and that copy number varies across strains for a subset of tRNAs.

Single-copy tRNAs can exist either as a non-selected tRNA variant mutated from a multicopy version or as an ancestral tRNA variant that is conserved, possibly for some specialized purpose [30]. Although multiple-copy tRNAs are more likely to be transcribed compared to tRNAs that exist in a single copy ( [20]; Supplementary Figure S2), we next examined whether some single-copy tRNAs are conserved and may serve specialized functions. One such example is the brain-enriched tRNA-Arg-TCT-4, originally identified in the human genome [28]. A slight variant of this tRNA exists in the mouse reference genome, also annotated as tRNA-Arg-TCT-4, differing from the human tRNA sequence by a single base (Supplementary Figure S3). We confirmed that the exact sequence of this human brain-enriched tRNA is present in the genomes of all mouse strains (Supplementary Figure S3), except for the reference strain (C57BL/6 J), suggesting a recent change in this tRNA in the C57BL/6 J strain [28]. As in the human genome, this tRNA exists as a single copy in all mouse strain assemblies. These analyses indicate that this brain-specific tRNA is highly conserved and remains single-copy, suggesting that specialized tRNAs may be constrained to maintain sequence fidelity and copy number in order to preserve their specialized functions and avoid misregulation or excessive transcript production.

**Fig. 3:**
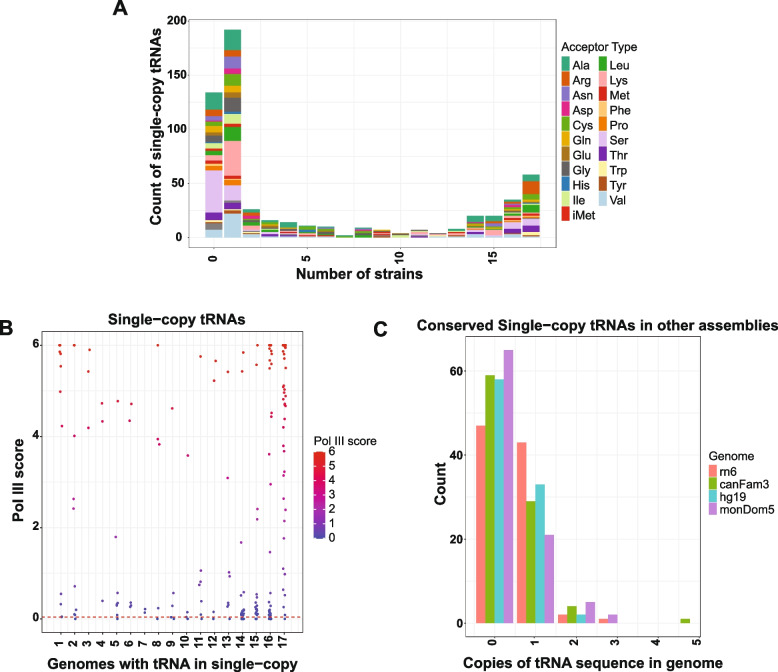
Divergence and conservation of single-copy tRNA genes across mouse strains: **A** Histogram showing the number of mouse strains in which each unique tRNA transcript, present in at least one strain, is maintained as a single-copy locus. A value of “0” indicates transcripts that are either multicopy or absent across all strains. **B** Strip plot showing the number of mouse strains in which each reference strain tRNA is present as a single-copy locus plotted against its Pol III score. Only tRNAs present in the mm10 reference assembly are included. The horizontal red dashed line indicates the Pol III score of the brain-specific mouse tRNA sequence, which is not present in the reference genome due to a single-nucleotide substitution. Pol III score of single-copy tRNAs present in all 17 strains is significantly higher than the Pol III score of those present in less than 16 (p-value is 0.0002795; Mann Whitney U-test). **C** Histogram showing the distribution of tRNA gene copy numbers in rat, dog, human, and possum genomes for tRNA genes that are single-copy across more than 10 mouse strains

To identify additional candidate specialized single-copy tRNAs, we searched mouse genomes for high-scoring tRNAs that occur as a single copy in all or most strains. Despite the close evolutionary relationships among these strains, most single-copy tRNAs were strain-specific and transient, appearing as single-copy loci in only one strain (Fig. 3A). In contrast, we identified 93 tRNAs that are maintained as single-copy loci in at least 16 mouse genomes. The strong conservation of these tRNAs across strains suggests that they represent candidates for specialized single-copy tRNAs (Fig. 3A). All 93 single-copy tRNAs were orthologous to their copies in other genomes according to our cactus graph-derived ortholog sets (Table S3), indicating that each of them likely originated from their own single-copy ancestral tRNA loci. Analysis of RNA Pol III ChIP-seq data from the reference genome revealed that many single-copy tRNAs conserved across all mouse strains are actively transcribed, whereas tRNAs present in fewer genomes generally exhibited lower Pol III occupancy (Fig. 3B). These results indicate that highly conserved single-copy tRNAs represent functional, expressed genes, at least in liver tissue. The brain-enriched tRNA-Arg-TCT-4, which is conserved as a unique locus in all but one assembly, was not representative of this group, consistent with its lack of transcription in liver [43].

To further assess evolutionary conservation, we examined the copy number of these 93 mouse single-copy tRNAs in additional mammalian genomes, including rat, human, dog, and possum. Of these, 43 (46.2% %) remained single-copy in rat and 33 (35.4% %) were conserved as single-copy in human, while most others were either absent or present in multiple copies in these species (Fig. 3C). Single-copy tRNAs conserved only within mouse strains may therefore reflect species-specific specialization. Notably, 9 tRNAs were maintained as single-copy loci across at least 16 mouse strains as well as rat, human, dog, and possum (Table S4). With the exception of tRNA-Arg-TCT-4, all of these loci showed Pol III binding in mouse liver, suggesting that they represent a core set of essential tRNAs that have resisted duplication, loss, or structural change over evolutionary time.

### Orthologous tRNAs change even in closely related mouse strains

**Fig. 4:**
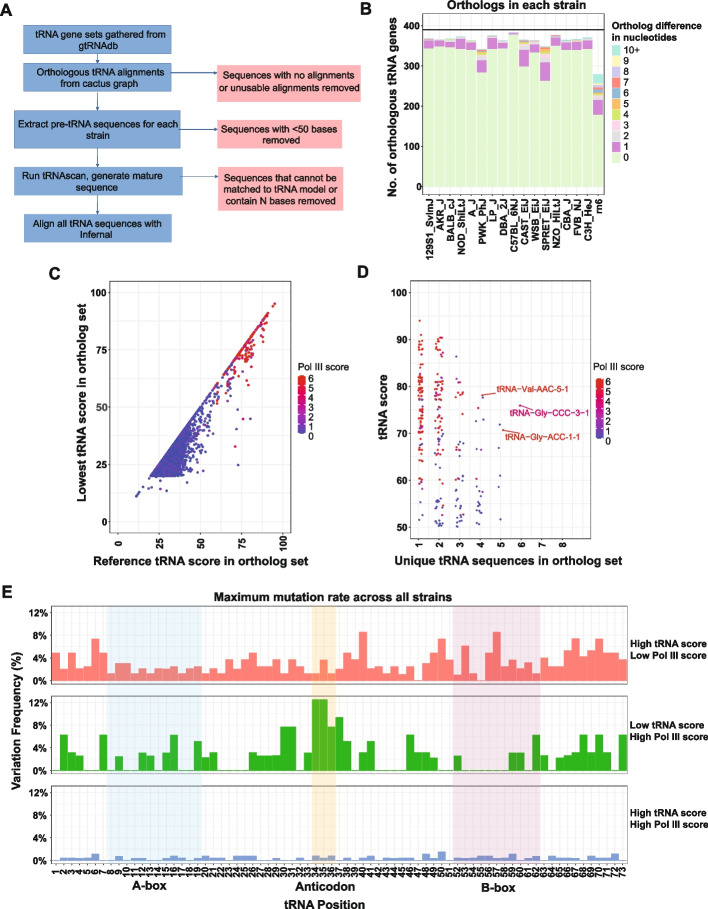
Sequence variation and Pol III occupancy across orthologous tRNAs in mouse strains: **A** Workflow diagram illustrating the generation of tRNA ortholog sets from mouse strain cactus graphs. Red squares indicate steps at which strain sequences are excluded from the final dataset, either due to missing orthologs or failure to identify or accurately reconstruct orthologous sequences. **B** Counts of tRNA orthologs corresponding to high-scoring reference mouse tRNAs across non-reference mouse strains and rat. Counts are colored according to the number of mismatches detected in each ortholog. **C** Comparison of reference tRNA scores with the minimum score observed among all orthologs for each tRNA or tRNA-like element. Data points are colored by Pol III score to indicate relative Pol III occupancy. **D** Strip plot showing the relationship between the number of unique sequence variants identified across mouse strains and the tRNA quality score, with points colored by Pol III score. **E** Bar plot showing maximum variation frequency (count of orthologous tRNAs with variant nucleotides/total tRNAs) across all strains for Sprinzl tRNA positions in high-scoring Pol III silent tRNAs (high tRNA score, low Pol III score), low-scoring Poll III bound tRNA-like elements (low tRNA score, high Pol III score), and high-scoring Pol III active tRNAs (high tRNA and Pol III score)

We next examined tRNA gene orthologs across mouse strains to identify evolutionarily conserved and strain-specific tRNA loci. Because tRNAs often exist in multiple identical genomic copies, orthologs cannot be inferred solely from sequence similarity, as is typically done for protein-coding genes. To overcome this limitation, we utilized previously published whole-genome alignments generated using the Cactus graph framework [35]. These alignments include the genomes of 17 assembled mouse strains and the rat genome as an outgroup, providing high-resolution correspondence across orthologous genomic regions. By extracting genome alignments corresponding to annotated tRNAs and tRNA-like elements, we assembled a comprehensive set of orthologous tRNA loci and tRNA-like elements shared among all mouse strains and rat (Fig. 4A, Table S3). For each tRNA gene, these ortholog sets identify its corresponding ortholog in each mouse strain and in rat, when such an ortholog could be found. Using this approach, we identified orthologous tRNAs or tRNA-like elements for more than 88% of all high-scoring tRNAs across strains, with the remaining loci absent due to genomic variation or incomplete assemblies. Notably, we observed substantial sequence divergence in high-scoring tRNAs even among these closely related strains: 5 tRNAs (1.3%) differed in sequence in C57BL/6NJ, the strain most closely related to the reference genome, whereas up to 32% of tRNAs showed sequence changes in the most divergent strain, SPRET/EiJ (derived from *Mus spretus)* (Fig. 4B; Supplementary Figure S4). The rat genome exhibited the greatest divergence, with only 178 (45%) of tRNAs identical to their mouse orthologs and 58 (15%) differing by more than one nucleotide. These findings reveal that, despite the strong functional constraints on tRNA genes, there is extensive sequence variation at individual loci, and it scales with evolutionary distance.

These differences between related genomes also include tRNAs for which an orthologous sequence can be identified but that appear to be non-functional tRNAs. Specifically, we found 3 tRNAs that are both high-scoring and transcriptionally active in the reference mouse genome but appear to have lost functionality in one or more strains (based on Pol III score) (Fig. 4C). Analysis of the specific sequence variations in these 3 tRNAs did not reveal shared changes in structural or regulatory elements, indicating that loss of Pol III signal in these strains is not associated with common alterations in core tRNA sequence features (Supplementary Figure S5). Moreover, while many tRNAs remain sequence-identical across all mouse strains or differ by only a single mutation, a subset accumulates multiple sequence changes. For example, some tRNA ortholog sets contain up to six distinct sequence variants across strains (Fig. 4D), including tRNAs actively transcribed by RNA Pol III in reference genome, with tRNA-Val-AAC-5–1, tRNA-Gly-CCC-3–1, and tRNA-Gly-ACC-1–1 all having more than 4 variants and being Pol III transcribed (Fig. 4D; Figure S6A–C). In rare cases, tRNA loci annotated in the reference genome display changes in decoder identity across mouse strains. For example, the locus annotated as tRNA-Gln-CTG-3–1 corresponds to tRNA-Gln-TTG-6 in four strains (Table S5). We also note that loci annotated as tRNA-Gly-ACC in the reference genome reflect annotation artifacts. Due to biochemical constraints imposed by ADAT-mediated editing, a tRNA-Gly-ACC decoder is not expected to occur in the mouse genome [44]. Consistent with this interpretation, these loci correspond instead to tRNA-Val isotype mismatches in three strains (Table S5). Together, these analyses reveal that tRNA loci, while generally highly conserved, can be dynamically evolving.

To assess how sequence conservation varies across individual tRNA positions, we compared high-scoring Pol III–bound tRNA genes (defined by combined tRNA and Pol III scores) with high-scoring tRNAs that are not Pol III bound and with low-scoring tRNAs that are Pol III bound (Fig. 4E; Supplementary Figure S7). We found that at nearly all positions (97%), transcribed high-scoring tRNAs exhibit greater sequence conservation than non-transcribed high-scoring tRNAs. In contrast, low-scoring but transcribed tRNA-like elements display a more complex pattern: overall they are more variable than high-scoring transcribed tRNAs, yet they contain a mixture of conserved and highly variable positions. Importantly, these patterns are not explained by recently inactivated tRNAs. Instead, these transcribed tRNA-like elements fail to form high-quality tRNA sequences in any mouse strain analyzed (Supplementary Figure S8). More than half of this gene set is serine derived, which is the most abundant SINE element in mice (Fig. 1B), suggesting that these are likely active SINE elements. The functional significance of these low-scoring Pol III transcribed tRNA-like elements remains unclear at this point. One tRNA-like gene that is known to be Pol III-transcribed is the BC1 non-coding RNA gene (here annotated as “tRX-Ala-NNN-120–1”), a brain-specific gene known to be derived from alanine tRNAs [45] and controls spine density and when knocked out leads to learning impairment in mice [46]. This gene, similar to tRNA-Arg-TCT-4, is not detected as expressed in our dataset because brain transcription was not assayed. However, this group of low-scoring, Pol III–transcribed tRNA-like loci may include additional tRNA-derived non-coding RNA genes with regulatory functions.

### Gain and loss of tRNA genes occur in related mouse genomes

**Fig. 5:**
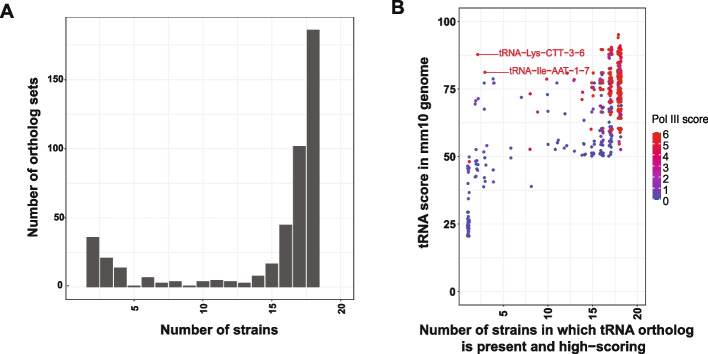
Strain-specific gain and loss of tRNA loci in mouse genomes: **A** Histogram showing the number of mouse strains represented in each predicted tRNA ortholog set. Only high-scoring tRNAs (score > 50) are included. **B** Strip plot comparing reference mm10 tRNA scores with the number of high-scoring orthologs per ortholog set. Data points are colored by Pol III score to indicate relative RNA Pol III occupancy

To investigate the gain and loss of tRNA genes across mouse strains, we next examined ortholog sets to identify loci that are either newly added or missing relative to the reference genome. For this analysis, we included high-scoring tRNAs represented in each ortholog set. We reasoned that tRNAs present in nearly all strains likely represent ancestral loci, whereas those absent in one or a few strains likely reflect recent deletions, and those present in only a small subset of strains represent recent insertions. Consistent with this prediction, the distribution of strain counts was bimodal, with most ortholog sets present in either one strain or all strains and relatively few represented in an intermediate number of strains (5–10) (Fig. 5A). Using this approach, we identified 2 tRNAs that are present in the reference genome and Pol III transcribed (tRNA-Lys-CTT-3–6 and tRNA-Ile-AAT-1–7) but not conserved in many strains, indicating ongoing turnover within the transcribed tRNA gene repertoire (Fig. 5B).

### Gene conversion in tRNAs and tRNA-like elements

Previous work on tRNA concerted evolution [47] raised the question of gene conversion that could be occurring in closely related tRNAs. To find such potential events, we searched pairs of strain genomes for tRNAs that change from one multicopy form to another. Although this cannot detect all conversion events, such precise base substitutions are highly unlikely to occur through random mutation alone, making them strong candidates for gene conversion. Using this strategy, we identified a single high-confidence case: the tRNA-Gln-CTG-2–1 in the reference genome, whose ortholog in the CAST/EiJ strain corresponds to tRNA-Gln-CTG-3 (Supplementary Figure S9). These two tRNAs differ by only a single nucleotide, consistent with a recent conversion event between highly similar loci. While this analysis cannot unambiguously determine the directionality of conversion, the tRNA-Gln-CTG-2–1 sequence is retained in all strains except CAST/EiJ, suggesting that the conversion likely occurred in the CAST/EiJ genome.

Expanding this approach to include low-scoring tRNA-like elements revealed additional evidence of gene conversion. Although we cannot exclude the possibility that some signals arise from assembly artifacts associated with repetitive regions, this broader analysis identified not only isolated conversion events but also recurrent, large-scale conversions, in which the same tRNA-like sequence was repeatedly replaced by a specific alternative sequence across multiple strains. We identified 9 distinct tRNA gene sequence pairs that appear to have undergone conversion ten or more times, where one tRNA gene sequence consistently replaces another (Table S6). Although these tRNA-like elements are unlikely to be transcriptionally active or functional, their recurrent sequence exchanges and similarity in size and sequence show that this type of gene conversion is possible and suggest that they may be subject to the same gene conversion mechanisms shaping active tRNA loci.

## Discussion

While tRNA evolution has been studied across kingdoms, genera, and species [10, 48–52], the extent to which mammalian tRNA genes evolve over relatively short timescales has not been examined systematically. Here, using a combination of covariance scoring and RNA Pol III ChIP-seq as a readout of active tRNAs, we provide a systematic analysis of tRNA gene conservation, diversification, copy number variation, and concerted evolution across 17 mouse strain genomes. While these mouse strain assemblies are not as high-quality as the reference assembly and may contain assembly errors, after the removal of sequences containing ambiguous bases (Ns) we believe them to be generally accurate for tRNAs. We find that although tRNA genes are among the most conserved features of mammalian genomes [4–6], significant variability exists in both sequence and copy number, even among these closely related strains. This indicates that the tRNA complement is not as static as previously assumed, but rather subject to rapid evolutionary changes that may have functional consequences for translation and other gene regulatory processes.

By integrating sequence-based covariance scores [14] with Pol III ChIP-seq data [36], we demonstrate that covariance scoring provides a useful and reasonably accurate predictor of transcriptionally active tRNAs, barring a few cases that can be attributed to tissue specificity, as the Pol III-ChIP-seq data were derived from liver tissues. Accordingly, some high-scoring tRNAs with low Pol III occupancy likely reflect tissue-specific Pol III engagement. For example, the highly conserved tRNA-Arg-TCT-4 gene shows strong Pol III occupancy in neuronal tissues and little to no occupancy in liver. These findings highlight the importance of integrating structural predictions with tissue- and condition-specific transcriptional data to fully capture the functional repertoire of tRNA genes. Additionally, although Pol III binding indicates that a locus is transcriptionally engaged, Pol III occupancy alone does not imply that the resulting transcript constitutes a stable or functional decoding tRNA. Structural and biochemical constraints [44, 53, 54], including anticodon identity and compatibility with known modification pathways, may limit tRNA stability and function even at Pol III–bound loci.

We find that tRNA copy numbers are largely stable across mouse strains, consistent with the strong evolutionary constraints on tRNA dosage and decoding balance. Nevertheless, some tRNAs exhibit copy number differences of up to 5 copies between strains. Although tRNAs are often considered redundant elements in translational regulation, duplicated to increase the number that can be transcribed, prior work has demonstrated that certain single-copy, uniquely occurring tRNAs can carry specialized functions [28]. In our analysis, we found that while many single-copy tRNAs are not conserved across mouse strains, a subset remains conserved and thus represents strong candidates for specialized roles. Interestingly, these conserved single-copy tRNAs generally do not follow the pattern of the well-characterized brain-specific tRNA but instead show RNA Pol III ChIP-seq signatures indicative of liver-specific Pol III engagement. The evolutionary pressure to maintain these tRNAs as single, unique copies is not yet clear, but their conservation suggests functional importance. That all but one of these conserved single-copy tRNAs are expressed in the liver does suggest that tissue specificity is not the primary form of specialization. Possible roles of these tRNAs include contributing to the translation of a restricted set of genes, functioning in a conditionally regulated manner where they are expressed or silenced depending on context, or serving as a source of regulatory tRNA fragments with distinct biological functions.

Our ortholog analyses revealed that actively transcribed tRNAs can be gained, lost, or altered in sequence even among closely related strains. Some transcribed orthologous sets showed multiple sequence variants, while others lost predicted structural integrity despite being transcribed in the reference genome. A small number of tRNAs even change in decoder or acceptor type. We also uncovered high conservation across all positions in transcribed tRNAs in mouse strains, with non-transcribed but otherwise high-quality tRNAs showing much higher variation, suggesting that these are generally silent tRNAs and tRNAs only transcribed in some species like tRNA-Arg-TCT-4 are rare. Finally, our search for tRNA concerted evolution showed some signs of gene conversion, which is consistent with the idea of concerted evolution proposed in yeast [32]. While genes that mutate to a novel form and then revert cannot be detected, we do find signs of tRNAs and, particularly, tRNA-like elements undergoing gene conversion.

## Conclusion

Together, these findings underscore that while the tRNA pool is highly conserved overall, subsets of tRNAs are evolving rapidly, with potential implications for translational efficiency, codon bias adaptation, and phenotypic diversity across strains. These results provide a resource for understanding the evolution of the tRNA repertoire in mammals and highlight the need to consider tRNA variation in studies of gene regulation, genome evolution, and strain-specific phenotypes. This work also identified a set of conserved and functionally significant tRNAs that represent promising targets for experimental manipulation to elucidate their biological roles in the genome.

## Supplementary Information

Supplementary Material 1: Supplementary Figures S1-S9.

Supplementary Material 2: Supplementary Table S1. Table listing tRNA and tRNA-like sequences identified across all mouse strains. Amino acid identity, anticodon, and tRNA scores were predicted using tRNAscan-SE. Sequence names correspond to mm10 reference annotations where available, and new identifiers were assigned to sequences not present in the mm10 reference set. Copy numbers for each tRNA are reported for all mouse strains and for the rn6 rat genome.

Supplementary Material 3: Supplementary Table S2. Table summarizing counts of high-scoring and low-scoring tRNA genes across mouse strain genomes, with corresponding “high confidence” tRNA counts from gtRNAdb included for comparison.

Supplementary Material 4: Supplementary Table S3. Table listing ortholog sets for tRNAs and tRNA-like elements. “NA” indicates cases in which no orthologous tRNA-like element was identified. tRNA names correspond to sequences listed in Table S1. Where available, ortholog set names include the locus identifier from the mm10 reference genome; otherwise, locus identifiers from non-reference strains are used.

Supplementary Material 5: Supplementary Table S4. List of tRNAs present as single-copy loci in more than 15 mouse strain genomes and as single-copy loci in the rn6 rat, canFam3 dog, hg19 human, and monDom5 opossum genomes, along with their corresponding Pol III scores.

Supplementary Material 6: Supplementary Table S5. List of all high-scoring and transcriptionally active (based on RNA Poll III score) ortholog pairs relative to the reference genome that show a change in anticodon identity.

Supplementary Material 7: Supplementary Table S6. List of frequencies of all inferred gene conversion events involving tRNAs and tRNA-like elements for each gene pair across all strains.

## Data Availability

Genome sequences were obtained from UCSC mouse strain assembly hub ([https://hgdownload.soe.ucsc.edu/hubs/mouseStrains/hubIndex.html]), The cactus alignment is from Mouse cactus strain hub [http://hgdownload.soe.ucsc.edu/hubs/mouseStrains/mouseStrains_1509.hal] (http://hgdownload.soe.ucsc.edu/hubs/mouseStrains/mouseStrains_1509.hal) RNA Pol III ChIP-Seq data is from NCBI SRA RPC4: ([https://trace.ncbi.nlm.nih.gov/Traces/?view=study&acc=SRP148448]) POLR3D: ([https://trace.ncbi.nlm.nih.gov/Traces/?study=SRP422530]) POLR3C, POLR3GL, POLR3G: (https://trace.ncbi.nlm.nih.gov/Traces/?study=SRP243597).
