# Supplementary material for: Comparative analysis of tRNAs across mouse strains reveals gene-specific conservation, divergence, and copy number variation
Source: BMC Genomics. 2026 Mar 26;27:464. doi: 10.1186/s12864-026-12787-8 (PMC13169703; doi:10.1186/s12864-026-12787-8)
Supplement: Supplementary file 1 — Supplementary Material 1: Supplementary Figures S1-S9. [file 12864_2026_12787_MOESM1_ESM.pdf]

Supplementary Figure S1

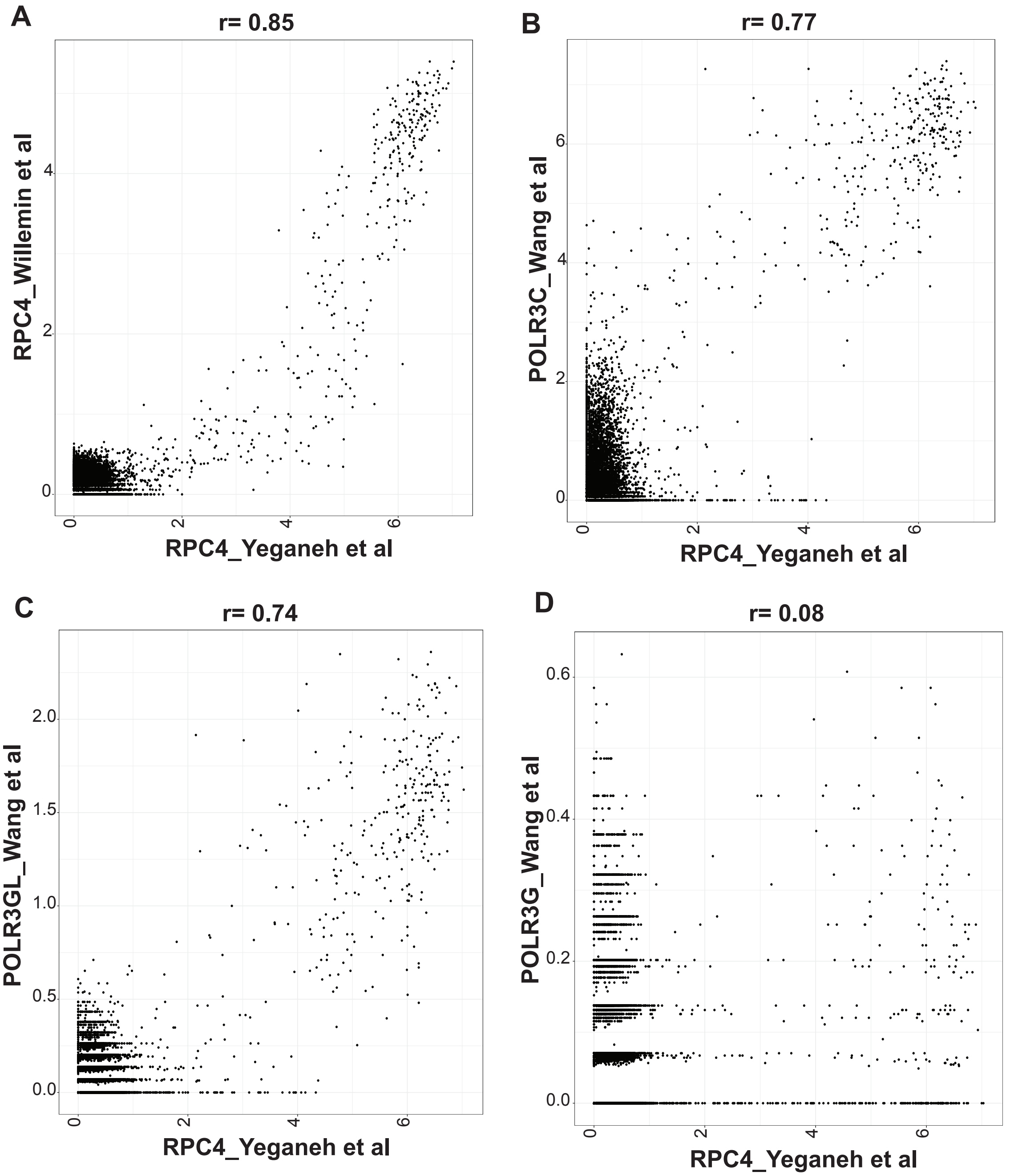

Supplementary Figure S1: Comparison of RNA Pol III ChIP-seq enrichment across all annotated tRNAs and tRNA-like elements using previously published datasets, including the Yeganeh et al. dataset used in this study to derive Pol III scores. For all panels, Pol III enrichment is calculated as the log2 ratio of Pol III ChIP-seq read counts to matched control read counts, and Pearson correlation coefficients are shown. **(A)** Anti- RPC4 (also called POLR3D) ChIP-seq (SRR23380860) normalized to control (SRR23380845) from Willemin et al. compared to Pol III enrichment values from Yeganeh et al. **(B–D)** Comparisons of POLR3C (SRR10918010), POLR3GL (SRR10918013), and POLR3G (SRR10918011) ChIP-seq datasets, each normalized to control (SRR10918014), against Pol III enrichment values from Yeganeh et al.

Supplementary Figure S2

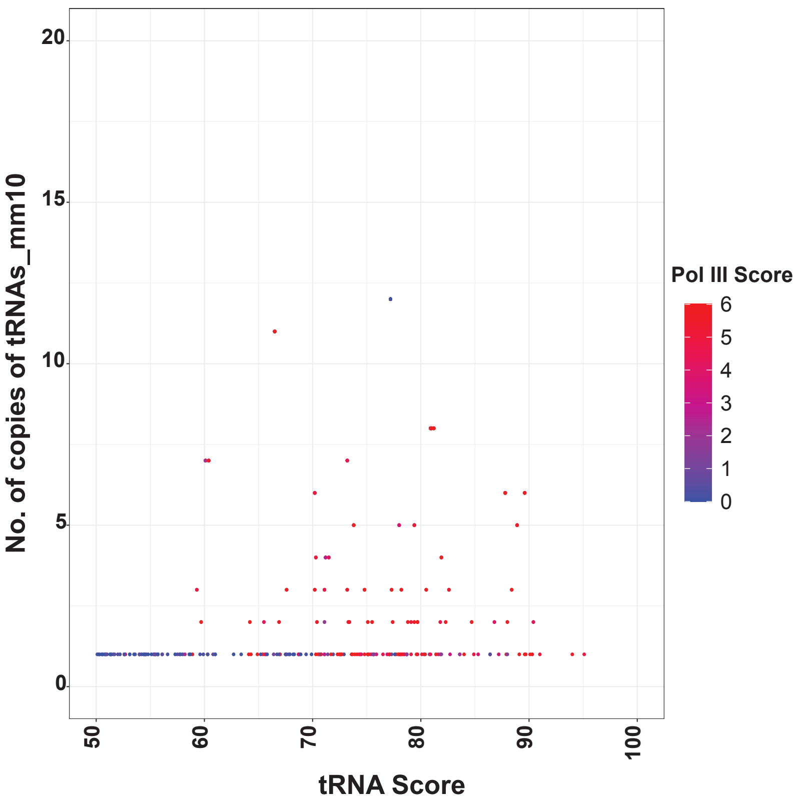

Supplementary Figure S2: Comparison of tRNA gene copy number with tRNA score and calculated Pol III score for high-scoring tRNAs in the mm10 reference genome.

Supplementary Figure S3

mm10	tRNA-Arg-TCT-4-1	GTCTCTGTGGCGCAATGGACGAGCGCGCTGGACTTCTAATCCAGATTGTTCTGGGTTCGAGTCCCGGCAGAGATGCCA
hg19	tRNA-Arg-TCT-4-1	GTCTCTGTGGCGCAATGGACGAGCGCGCTGGACTTCTAATCCAGAGGTTCCGGGTTCGAGTCCCGGCAGAGATGCCA
SPRET_EiJ	tRNA-Arg-TCT-4-1	GTCTCTGTGGCGCAATGGACGAGCGCGCTGGACTTCTAATCCAGAGGTTCCGGGTTCGAGTCCCGGCAGAGATGCCA
WSB_EiJ	tRNA-Arg-TCT-4-1	GTCTCTGTGGCGCAATGGACGAGCGCGCTGGACTTCTAATCCAGAGGTTCCGGGTTCGAGTCCCGGCAGAGATGCCA
129S1_SvImJ	tRNA-Arg-TCT-4-1	GTCTCTGTGGCGCAATGGACGAGCGCGCTGGACTTCTAATCCAGAGGTTCCGGGTTCGAGTCCCGGCAGAGATGCCA
DBA_2J	tRNA-Arg-TCT-4-1	GTCTCTGTGGCGCAATGGACGAGCGCGCTGGACTTCTAATCCAGAGGTTCCGGGTTCGAGTCCCGGCAGAGATGCCA
C3H_HeJ	tRNA-Arg-TCT-4-1	GTCTCTGTGGCGCAATGGACGAGCGCGCTGGACTTCTAATCCAGAGGTTCCGGGTTCGAGTCCCGGCAGAGATGCCA
CBA_J	tRNA-Arg-TCT-4-1	GTCTCTGTGGCGCAATGGACGAGCGCGCTGGACTTCTAATCCAGAGGTTCCGGGTTCGAGTCCCGGCAGAGATGCCA
NZO_HlLtJ	tRNA-Arg-TCT-4-1	GTCTCTGTGGCGCAATGGACGAGCGCGCTGGACTTCTAATCCAGAGGTTCCGGGTTCGAGTCCCGGCAGAGATGCCA
AKR_J	tRNA-Arg-TCT-4-1	GTCTCTGTGGCGCAATGGACGAGCGCGCTGGACTTCTAATCCAGAGGTTCCGGGTTCGAGTCCCGGCAGAGATGCCA
A_J	tRNA-Arg-TCT-4-1	GTCTCTGTGGCGCAATGGACGAGCGCGCTGGACTTCTAATCCAGAGGTTCCGGGTTCGAGTCCCGGCAGAGATGCCA
CAST_EiJ	tRNA-Arg-TCT-4-1	GTCTCTGTGGCGCAATGGACGAGCGCGCTGGACTTCTAATCCAGAGGTTCCGGGTTCGAGTCCCGGCAGAGATGCCA
NOD_ShiLtJ	tRNA-Arg-TCT-4-1	GTCTCTGTGGCGCAATGGACGAGCGCGCTGGACTTCTAATCCAGAGGTTCCGGGTTCGAGTCCCGGCAGAGATGCCA
BALB_cJ	tRNA-Arg-TCT-4-1	GTCTCTGTGGCGCAATGGACGAGCGCGCTGGACTTCTAATCCAGAGGTTCCGGGTTCGAGTCCCGGCAGAGATGCCA
LP_J	tRNA-Arg-TCT-4-1	GTCTCTGTGGCGCAATGGACGAGCGCGCTGGACTTCTAATCCAGAGGTTCCGGGTTCGAGTCCCGGCAGAGATGCCA
PWK_PhJ	tRNA-Arg-TCT-4-1	GTCTCTGTGGCGCAATGGACGAGCGCGCTGGACTTCTAATCCAGAGGTTCCGGGTTCGAGTCCCGGCAGAGATGCCA
FVB_NJ	tRNA-Arg-TCT-4-1	GTCTCTGTGGCGCAATGGACGAGCGCGCTGGACTTCTAATCCAGAGGTTCCGGGTTCGAGTCCCGGCAGAGATGCCA
C57BL_6NJ	tRNA-Arg-TCT-4-1	GTCTCTGTGGCGCAATGGACGAGCGCGCTGGACTTCTAATCCAGAGGTTCCGGGTTCGAGTCCCGGCAGAGATGCCA
rn6	tRNA-Arg-TCT-4-1	GTCTCTGTGGCGCAATGGACGAGCGCGCTGGACTTCTAATCCAGAGGTTCCGGGTTCGAGTCCCGGCAGAGATGCCA

Supplementary Figure S3: Sequence alignment of the tRNA-Arg-TCT-4 locus across the human hg19 genome, the mouse mm10 reference genome, and additional mouse strain genomes. The nucleotide position exhibiting variation is highlighted in yellow, with the mm10-specific substitution indicated in red.

Supplementary Figure S4

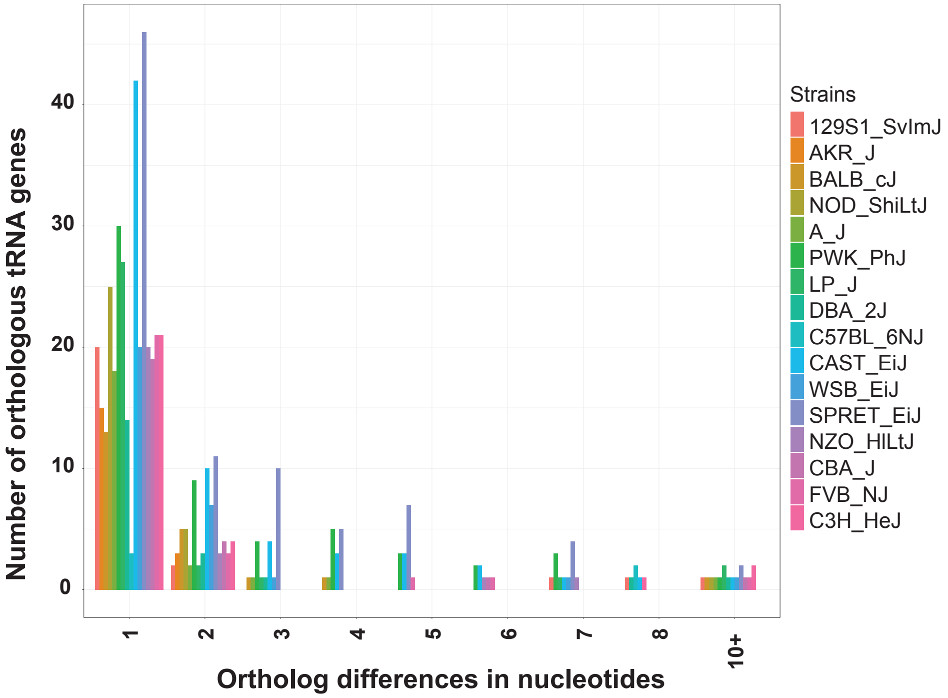

Supplementary Figure S4: Histogram showing the distribution of orthologs corresponding to high-scoring reference mouse tRNAs across non-reference mouse strains and rat (also shown in Figure 4B).

Supplementary Figure S5

mm10	tRNA-Ala-AGC-10	GGGGGATTAGCTCAAATGGTAGAGCGCTCGCTTAGCATGCAAGAGGTAGTGGGATCGATGCCCACATCCTCCACCA
SPRET_EiJ	tRNA-Ala-AGC-5	GGGGGATTAGCTCAAATGGTAGAGCGCTCGCTTAGCATGCGAGAGGTAGTGGGATCGATGCCCACATCCTCCACCA
WSB_EiJ	tRNA-Ala-AGC-10	GGGGGATTAGCTCAAATGGTAGAGCGCTCGCTTAGCATGCAAGAGGTAGTGGGATCGATGCCCACATCCTCCACCA
129S1_SvImJ	tRNA-Ala-AGC-10_C43G	GGGGGATTAGCTCAAATGGTAGAGCGCTCGCTTAGCATGCAACAGGTAGTGGGATCGATGCCCACATCCTCCACCA
NZO_HlLtJ	tRNA-Ala-AGC-10_C43G	GGGGGATTAGCTCAAATGGTAGAGCGCTCGCTTAGCATGCAACAGGTAGTGGGATCGATGCCCACATCCTCCACCA
A_J	tRNA-Ala-AGC-10_C43G	GGGGGATTAGCTCAAATGGTAGAGCGCTCGCTTAGCATGCAACAGGTAGTGGGATCGATGCCCACATCCTCCACCA
CAST_EiJ	tRNA-Ala-AGC-5	GGGGGATTAGCTCAAATGGTAGAGCGCTCGCTTAGCATGCGAGAGGTAGTGGGATCGATGCCCACATCCTCCACCA
NOD_ShiLtJ	tRNA-Ala-AGC-5	GGGGGATTAGCTCAAATGGTAGAGCGCTCGCTTAGCATGCGAGAGGTAGTGGGATCGATGCCCACATCCTCCACCA
BALB_cJ	tRNA-Ala-AGC-10_C43G	GGGGGATTAGCTCAAATGGTAGAGCGCTCGCTTAGCATGCAACAGGTAGTGGGATCGATGCCCACATCCTCCACCA
LP_J	tRNA-Ala-AGC-5	GGGGGATTAGCTCAAATGGTAGAGCGCTCGCTTAGCATGCGAGAGGTAGTGGGATCGATGCCCACATCCTCCACCA
PWK_PhJ	tRNA-Ala-AGC-5	GGGGGATTAGCTCAAATGGTAGAGCGCTCGCTTAGCATGCGAGAGGTAGTGGGATCGATGCCCACATCCTCCACCA
FVB_NJ	tRNA-Ala-AGC-10_C43G	GGGGGATTAGCTCAAATGGTAGAGCGCTCGCTTAGCATGCAACAGGTAGTGGGATCGATGCCCACATCCTCCACCA
C57BL_6NJ	tRNA-Ala-AGC-10	GGGGGATTAGCTCAAATGGTAGAGCGCTCGCTTAGCATGCAAGAGGTAGTGGGATCGATGCCCACATCCTCCACCA

A box Anticodon B box

mm10	tRNA-Gln-CTG-3	GGTTCCATGGTGTAATGGTTAGCACTCTGGACTCTGAATCCAGCGATCCGAGTTCAAATCTCGGTGGGACCTCCA
SPRET_EiJ	tRNA-Gln-CTG-3	GGTTCCATGGTGTAATGGTTAGCACTCTGGACTCTGAATCCAGCGATCCGAGTTCAAATCTCGGTGGGACCTCCA
WSB_EiJ	tRNA-Gln-CTG-3	GGTTCCATGGTGTAATGGTTAGCACTCTGGACTCTGAATCCAGCGATCCGAGTTCAAATCTCGGTGGGACCTCCA
129S1_SvImJ	tRNA-Gln-TTG-6	GTTTCCATGGTGTAATGGTTGGCACTCTGGACTTTGAATCCAGCAATCCAAGTTCAAGTCTCTGTGGGACCTCCA
DBA_2J	tRNA-Gln-TTG-6	GTTTCCATGGTGTAATGGTTGGCACTCTGGACTTTGAATCCAGCAATCCAAGTTCAAGTCTCTGTGGGACCTCCA
C3H_HeJ	tRNA-Gln-TTG-6	GTTTCCATGGTGTAATGGTTGGCACTCTGGACTTTGAATCCAGCAATCCAAGTTCAAGTCTCTGTGGGACCTCCA
CBA_J	tRNA-Gln-CTG-3	GGTTCCATGGTGTAATGGTTAGCACTCTGGACTCTGAATCCAGCGATCCGAGTTCAAATCTCGGTGGGACCTCCA
NZO_HlLtJ	tRNA-Gln-CTG-3	GGTTCCATGGTGTAATGGTTAGCACTCTGGACTCTGAATCCAGCGATCCGAGTTCAAATCTCGGTGGGACCTCCA
AKR_J	tRNA-Gln-CTG-3	GGTTCCATGGTGTAATGGTTAGCACTCTGGACTCTGAATCCAGCGATCCGAGTTCAAATCTCGGTGGGACCTCCA
A_J	tRNA-Gln-CTG-3	GGTTCCATGGTGTAATGGTTAGCACTCTGGACTCTGAATCCAGCGATCCGAGTTCAAATCTCGGTGGGACCTCCA
CAST_EiJ	tRNA-Gln-CTG-3	GGTTCCATGGTGTAATGGTTAGCACTCTGGACTCTGAATCCAGCGATCCGAGTTCAAATCTCGGTGGGACCTCCA
NOD_ShiLtJ	tRNA-Gln-CTG-3	GGTTCCATGGTGTAATGGTTAGCACTCTGGACTCTGAATCCAGCGATCCGAGTTCAAATCTCGGTGGGACCTCCA
BALB_cJ	tRNA-Gln-CTG-3	GGTTCCATGGTGTAATGGTTAGCACTCTGGACTCTGAATCCAGCGATCCGAGTTCAAATCTCGGTGGGACCTCCA
LP_J	tRNA-Gln-CTG-3	GGTTCCATGGTGTAATGGTTAGCACTCTGGACTCTGAATCCAGCGATCCGAGTTCAAATCTCGGTGGGACCTCCA
PWK_PhJ	tRNA-Gln-CTG-3	GGTTCCATGGTGTAATGGTTAGCACTCTGGACTCTGAATCCAGCGATCCGAGTTCAAATCTCGGTGGGACCTCCA
FVB_NJ	tRNA-Gln-CTG-3	GGTTCCATGGTGTAATGGTTAGCACTCTGGACTCTGAATCCAGCGATCCGAGTTCAAATCTCGGTGGGACCTCCA
C57BL_6NJ	tRNA-Gln-TTG-6	GTTTCCATGGTGTAATGGTTGGCACTCTGGACTTTGAATCCAGCAATCCAAGTTCAAGTCTCTGTGGGACCTCCA
rn6	tRNA-Gln-CTG-3	GGTTCCATGGTGTAATGGTTAGCACTCTGGACTCTGAATCCAGCGATCCGAGTTCAAATCTCGGTGGGACCTCCA

A box Anticodon B box

mm10	tRNA-Gly-CCC-3	GCATTGGTGGTTCAATGGTAGAATTCTCGCCTCCCACGCGGGTGACCCGGGTTCGATTCCCGGCCAATGCACCA
SPRET_EiJ	tRNA-Gly-CCC-3	GCATTGGTGGTTCAATGGTAGAATTCTCGCCTCCCACGCGGGTGACCCGGGTTCGATTCCCGGCCAATGCACCA
WSB_EiJ	tRNA-Gly-CCC-3	GCATTGGTGGTTCAATGGTAGAATTCTCGCCTCCCACGCGGGTGACCCGGGTTCGATTCCCGGCCAATGCACCA
129S1_SvImJ	tRNA-Gly-CCC-3	GCATTGGTGGTTCAATGGTAGAATTCTCGCCTCCCACGCGGGTGACCCGGGTTCGATTCCCGGCCAATGCACCA
DBA_2J	tRNA-Gly-CCC-3	GCATTGGTGGTTCAATGGTAGAATTCTCGCCTCCCACGCGGGTGACCCGGGTTCGATTCCCGGCCAATGCACCA
C3H_HeJ	tRNA-Gly-CCC-3_U32C	GCATTGGTGGTTCAATGGTAGAATTCTCGCTTCCCACGCGGGTGACCCGGGTTCGATTCCCGGCCAATGCACCA
CBA_J	tRNA-Gly-CCC-3_U32C	GCATTGGTGGTTCAATGGTAGAATTCTCGCTTCCCACGCGGGTGACCCGGGTTCGATTCCCGGCCAATGCACCA
NZO_HlLtJ	tRNA-Gly-CCC-3_U38C_U50C	GCATTGGTGGTTCAATGGTAGAATTCTCGCCTCCCATGCGGGTGACCTGGGTTCGATTCCCGGCCAATGCACCA
AKR_J	tRNA-Gly-CCC-3	GCATTGGTGGTTCAATGGTAGAATTCTCGCCTCCCACGCGGGTGACCCGGGTTCGATTCCCGGCCAATGCACCA
A_J	tRNA-Gly-CCC-3_U32C	GCATTGGTGGTTCAATGGTAGAATTCTCGCTTCCCACGCGGGTGACCCGGGTTCGATTCCCGGCCAATGCACCA
CAST_EiJ	tRNA-Gly-CCC-3_U29C	GCATTGGTGGTTCAATGGTAGAATTCTTGCCTCCCACGCGGGTGACCCGGGTTCGATTCCCGGCCAATGCACCA
NOD_ShiLtJ	alttRNA-Gly-CCC-6	GCATTGGTGGTTCAATGGTA---TTCTCGCTTCCCACGCGGGTGACCCGGGTTCGATTCCCGGCCAATGCACCA
BALB_cJ	tRNA-Gly-CCC-3_U38C_U50C	GCATTGGTGGTTCAATGGTAGAATTCTCGCCTCCCATGCGGGTGACCTGGGTTCGATTCCCGGCCAATGCACCA
LP_J	tRNA-Gly-CCC-3	GCATTGGTGGTTCAATGGTAGAATTCTCGCCTCCCACGCGGGTGACCCGGGTTCGATTCCCGGCCAATGCACCA
PWK_PhJ	tRNA-Gly-CCC-3_U3A	GCTTTGGTGGTTCAATGGTAGAATTCTCGCCTCCCACGCGGGTGACCCGGGTTCGATTCCCGGCCAATGCACCA
FVB_NJ	tRNA-Gly-CCC-3_U32C	GCATTGGTGGTTCAATGGTAGAATTCTCGCTTCCCACGCGGGTGACCCGGGTTCGATTCCCGGCCAATGCACCA
C57BL_6NJ	tRNA-Gly-CCC-3	GCATTGGTGGTTCAATGGTAGAATTCTCGCCTCCCACGCGGGTGACCCGGGTTCGATTCCCGGCCAATGCACCA
rn6	tRNA-Gly-CCC-3	GCATTGGTGGTTCAATGGTAGAATTCTCGCCTCCCACGCGGGTGACCCGGGTTCGATTCCCGGCCAATGCACCA

A box Anticodon B box

Supplementary Figure S5: Sequence alignments of three tRNAs that are transcriptionally active in the reference genome but exhibit low RNA polymerase III (Pol III) scores in other strains. Sequence variants are highlighted in yellow. Key tRNA gene features are indicated by colored boxes: A box (blue), anticodon (pink), and B box (green).

Supplementary Figure 6

A tRNA-Gly-ACC-1-1

mm10	GTTTCCGTAGTGTAGTGGTTAGCGCGTTCGCCTACCAAAGCGAAAGGTCCCCGGTTCGAAACCGGGCGGAAACACCA
FVB_NJ	GTTTCCGTAGTGTAGTGGTTAGCGCGTTCGCCTACCAAAGCGAAAGGTCCCCGGTTCGAAACCGGGCGGAAACACCA
NZO_HlLtJ	GTTTCCGTAGTGTAGTGGTTAGCACGTTCGCCTACCAAAGCGAAAGGTCCCCGGTTCGAAACCGGGCGGAAACACCA
BALB_cJ	GTTTCCGTAGTGTAGTGGTTAGCGCGTTCGCCTACCAAAGCGAAAGGTCCCCGGTTCGAAACCGGGCGGAAACACCA
CBA_J	GTTTCCGTAGTGTAGTGGTTAGCGCGTTCGCCTACCAAAGCGAAAGGTCCCCGGTTCGAAACCGGGCGGAAACACCA
C57BL_6NJ	GTTTCCGTAGTGTAGTGGTTAGCGCGTTCGCCTACCAAAGCGAAAGGTCCCCGGTTCGAAACCGGGCGGAAACACCA
DBA_2J	GTTTCCGTAGTGTAGTGGTTAGCGCGTTCGCCTACCAAAGCGAAAGGTCCCCGGTTCGAAACCGGGCGGAAACACCA
CAST_EiJ	GTTTCCGTAGTGTAGTGGTTAGCGCGTTCGCCTACCAAAGCGAAAGGTCCCCGGTTCGAAACCGGGCGGAAACACCA
SPRET_EiJ	GTTTCCGTAGTGTAGTGGTTATCACGTTCGCCTA-ACACGCGAAAGGTCCCCGGTTCAAAACCGGGCGGAAACACCA
C3H_HeJ	GTTTCCGTAGTGTAGTGGTTAGCGCGTTCGCCTACCAAAGCGAAAGGTCCCCGGTTCGAAACCGGGCGGAAACACCA
PWK_PhJ	GTTTCCGTAGTGTAGTGGTTATCACGTTCGCCTA-ACACGCGAAAGGTCCCCGGTTCGAAACCGGGCGGAAACACCA
NOD_ShiLtJ	GTTTCCGTAGTGTAGTGGTTAGCGCGTTCGCCTACCAAAGCGAAAGGTCCCCGGTTCGAAACCGGGCGGAAACACCA
X129S1_SvImJ	GTTTCCGTAGTGTAGTGGTTAGCGCGTTCGCCTACCAAAGCGAAAGGTCCCCGGTTCGAAACCGGGCGGAAACACCA
AKR_J	GTTTCCGTAGTGTAGTGGTTAGCACGTTCGCCTACCAAAGCGAAAGGTCCCCGGTTCGAAACCGGGCGGAAACACCA
A_J	GTTTCCGTAGTGTAGTGGTTAGCGCGTTCGCCTACCAAAGCGAAAGGTCCCCGGTTCGAAACCGGGCGGAAACACCA
WSB_EiJ	GTTTCCGTAGTGTAGTGGTTAGCACGTTCGCCTACCAAAGCGAAAGGTCCCCGGTACGAAACCGGGCGGAAACACCA
LP_J	GTTTCCGTAGTGTAGTGGTTAGCGCGTTCGCCTACCAAAGCGAAAGGTCCCCGGTTCGAAACCGGGCGGAAACACCA
rn6	GTTTCCGTAGTGTAGTGGTTATCACGTTCGCCTA-ACACGCGAAAGGTCCCCGGTTCGAAACCGGGCGGAAACACCA

B tRNA-Gly-CCC-3-1

mm10	GCATTGGTGGTTCAATGGTAGAATTCTCGCCTCCCACGCGGGTGACCCGGGTTCGATTCCCGGCCAATGCACCA
FVB_NJ	GCATTGGTGGTTCAATGGTAGAATTCTCGCTTCCCACGCGGGTGACCCGGGTTCGATTCCCGGCCAATGCACCA
NZO_HlLtJ	GCATTGGTGGTTCAATGGTAGAATTCTCGCCTCCCATGCGGGTGACCTGGGTTCGATTCCCGGCCAATGCACCA
BALB_cJ	GCATTGGTGGTTCAATGGTAGAATTCTCGCCTCCCATGCGGGTGACCTGGGTTCGATTCCCGGCCAATGCACCA
CBA_J	GCATTGGTGGTTCAATGGTAGAATTCTCGCTTCCCACGCGGGTGACCCGGGTTCGATTCCCGGCCAATGCACCA
C57BL_6NJ	GCATTGGTGGTTCAATGGTAGAATTCTCGCCTCCCACGCGGGTGACCCGGGTTCGATTCCCGGCCAATGCACCA
DBA_2J	GCATTGGTGGTTCAATGGTAGAATTCTCGCCTCCCACGCGGGTGACCCGGGTTCGATTCCCGGCCAATGCACCA
CAST_EiJ	GCATTGGTGGTTCAATGGTAGAATTCTTGCCTCCCACGCGGGTGACCCGGGTTCGATTCCCGGCCAATGCACCA
SPRET_EiJ	GCATTGGTGGTTCAATGGTAGAATTCTCGCCTCCCACGCGGGTGACCCGGGTTCGATTCCCGGCCAATGCACCA
C3H_HeJ	GCATTGGTGGTTCAATGGTAGAATTCTCGCTTCCCACGCGGGTGACCCGGGTTCGATTCCCGGCCAATGCACCA
PWK_PhJ	GCTTTGGTGGTTCAATGGTAGAATTCTCGCCTCCCACGCGGGTGACCCGGGTTCGATTCCCGGCCAATGCACCA
NOD_ShiLtJ	GCATTGGTGGTTCAATGGTA---TTCTCGCTTCCCACGCGGGTGACCCGGGTTCGATTCCCGGCCAATGCACCA
X129S1_SvImJ	GCATTGGTGGTTCAATGGTAGAATTCTCGCCTCCCACGCGGGTGACCCGGGTTCGATTCCCGGCCAATGCACCA
AKR_J	GCATTGGTGGTTCAATGGTAGAATTCTCGCCTCCCACGCGGGTGACCCGGGTTCGATTCCCGGCCAATGCACCA
A_J	GCATTGGTGGTTCAATGGTAGAATTCTCGCTTCCCACGCGGGTGACCCGGGTTCGATTCCCGGCCAATGCACCA
WSB_EiJ	GCATTGGTGGTTCAATGGTAGAATTCTCGCCTCCCACGCGGGTGACCCGGGTTCGATTCCCGGCCAATGCACCA
LP_J	GCATTGGTGGTTCAATGGTAGAATTCTCGCCTCCCACGCGGGTGACCCGGGTTCGATTCCCGGCCAATGCACCA
rn6	GCATTGGTGGTTCAATGGTAGAATTCTCGCCTCCCACGCGGGTGACCCGGGTTCGATTCCCGGCCAATGCACCA

C tRNA-Val-AAC-5-1

mm10	GTTTCCGTAGTGTAGTGGTTATCACATTCGCCTAACACGCGAAAGGTCCCCGGTTCGAAACCGGGCGGAAACACCA
FVB_NJ	GTTTCCGTAGTGTAGTGGTTATCACATTCGCCTAACACGCGAAAGGTCCCCGGTTCGAAACCGGGCGGAAACACCA
NZO_HlLtJ	GTTTCCGTAGTGTAGTGGTTATCACATTCGCCTAACACGCGAAAGGTCCCCGGTTCGAAACCGGGCGGAAACACCA
BALB_cJ	GTTTCCGTAGTGTAGTGGTTATCACATTCGCCTAACACGCGAAAGGTCCCCGGTTCGAAACCGGGCGGAAACACCA
CBA_J	GTTTCCGTAGTGTAGTGGTTATCACATTCGCCTAACACGCGAAAGGTCCCCGGTTCGAAACCGGGCGGAAACACCA
C57BL_6NJ	GTTTCCGTAGTGTAGTGGTTATCACATTCGCCTAACACGCGAAAGGTCCCCGGTTCGAAACCGGGCGGAAACACCA
DBA_2J	GTTTCCGTAGTGTAGTGGTTATCACATTCGCCTAACACGCGAAAGGTCCCCGGTTCGAAACCGGGCGGAAACACCA
CAST_EiJ	GTTTCCGTAGTGTAGTGGTTATCACATTCGCCTAACACGCGAAAGGTCCCCGGTTCGAAACCGGGCGGAAACACCA
SPRET_EiJ	GTTTCCGTAGTGTAGTGGTTATCACATTCGCCTAACACGCGAAAGGTCCCCGGTTCGAAACCGGGCGGAAACACCA
C3H_HeJ	GTTTCCGTAGTGTAGTGGTTATCACATTCGCCTAACACGCGAAAGGTCCCCGGTTCGAAACCGGGCGGAAACACCA
PWK_PhJ	GTTTCCGTAGTGTAGTGGTTATCACATTCGCCTAACACGCGAAAGGTCCCCGGTTCGAAACCGGGTGGAAACACCA
NOD_ShiLtJ	GTTTCCGTAGTGTAGTGGTTATCACATTCGCCTAACACGCGAAAGGTCCCCGGTTCGAAACCGGGCGGAAACACCA
X129S1_SvImJ	GTTTCCGTAGTGTAGTGGTTATCACATTCGCCTAACACGCGAAAGGTCCCCGGTTCGAAACCGGGCGGAAACACCA
AKR_J	GTTTCCGTAGTGTAGTGGTTATCACATTCGCCTAACACGCGAAAGGTCCCCGGTTCGAAACCGGGCGGAAACACCA
A_J	GTTTCCGTAGTGTAGTGGTTATCACATTCGCCTAACACGCGAAAGGTCCCCGGTTCGAAACCGGGCGGAAACACCA
WSB_EiJ	GTTTCCGTAGTGTAGTGGTTATCACATTCGCCTAACACGCGAAAGGTCCCCGGCCCGAAACCGGGCGGAAACACCA
LP_J	GTTTCCGTAGTGTAGTGGTTATCACATTCGCCTAACACGCGAAAGGTCTCCGGTTCGAAACCGGGCGGAAACACCA
rn6	GTTTCCGTAGTGTAGTGGTTATCACATTCGCCTAACACGCGAAAGGTCCCCGGTTCGAAACCGGGCGGGAACACCA

Supplementary Figure S6: Sequence alignments of ortholog sets for the tRNA-Gly-ACC-1-1, tRNA-Gly-CCC-3-1, and tRNA-Val-AAC-5-1 loci, showing the orthologous tRNA sequence from each strain. Sequences are color-coded such that identical sequences within each alignment share the same color, facilitating visualization of strain-specific variation.

Supplementary Figure S7

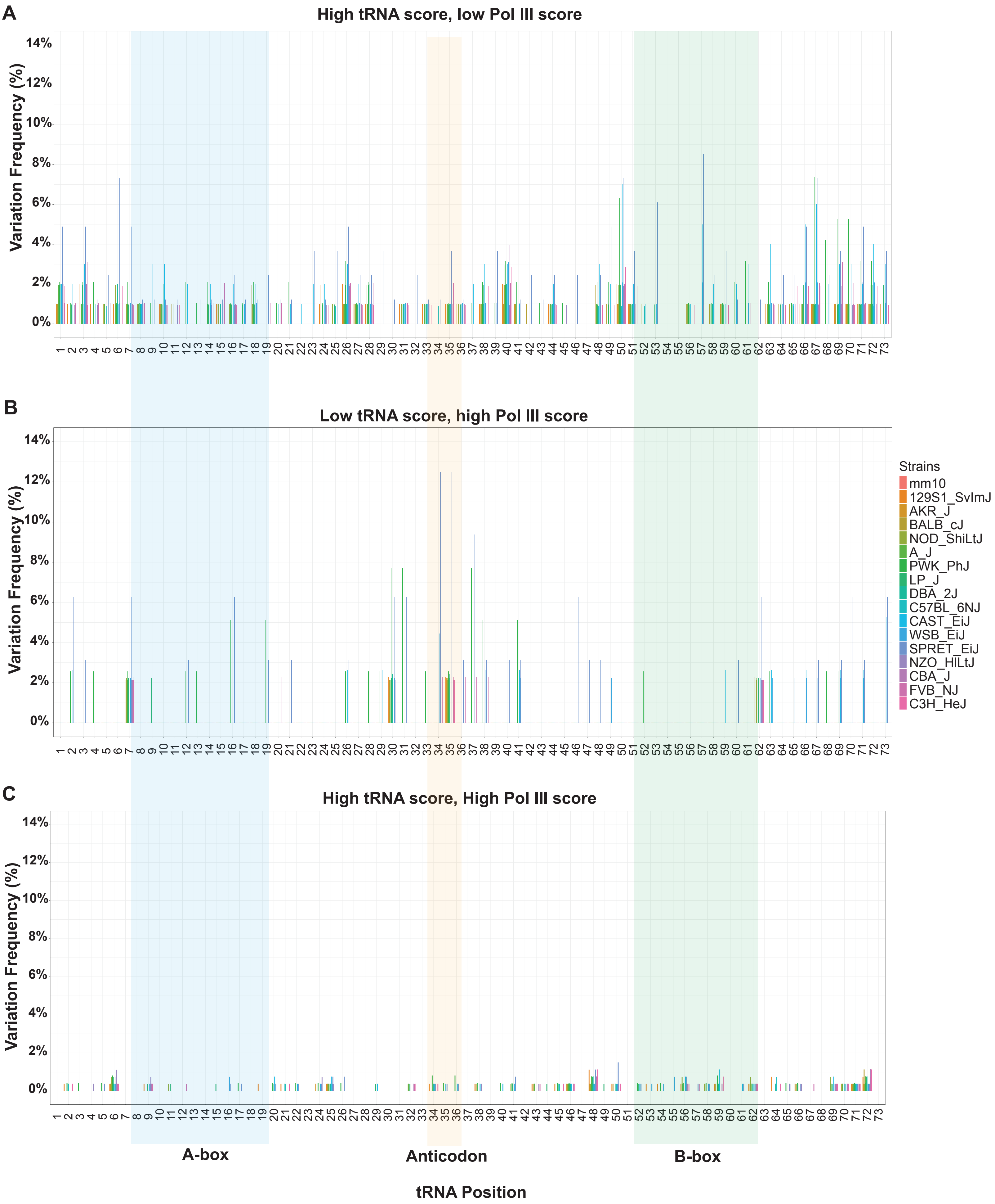

Supplementary Figure S7: Bar plot showing variation frequency (count of orthologous tRNAs with variant nucleotides / total tRNAs) for all strains in high-scoring Pol III silent tRNAs (**A**), low-scoring PolI III transcribed tRNA-like elements (**B**), and high-scoring Pol III transcribed tRNAs (**C**).

Supplementary Figure S8

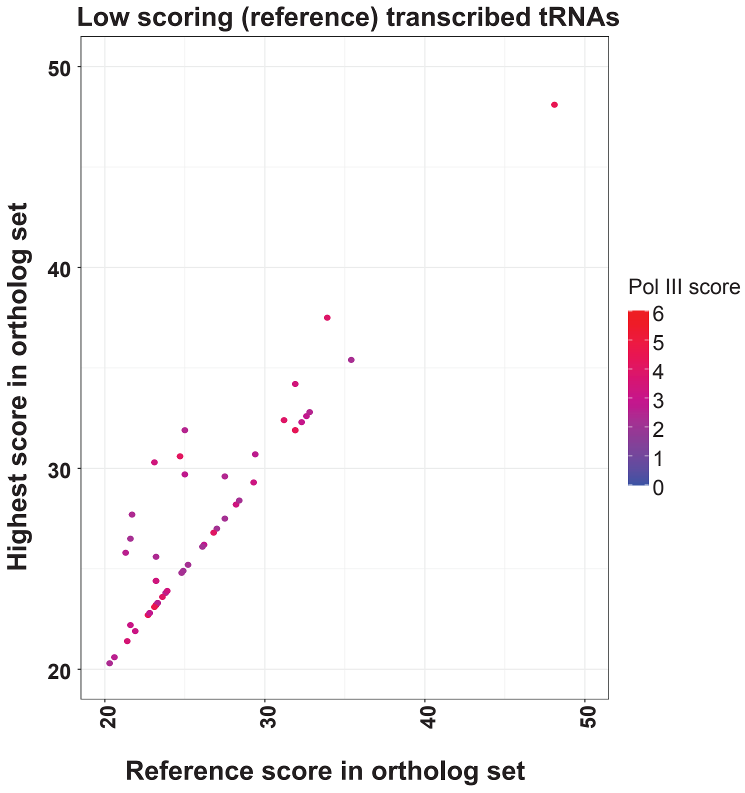

Supplementary Figure S8: Comparison of reference tRNA scores with the maximum score observed among all orthologs for each tRNA or tRNA-like element for all expressed Pol III tRNAs (Pol III score > 2) and tRNA score < 50 in reference. Data points are colored by Pol III score to indicate relative Pol III occupancy.

Supplementary Figure S9

```
mm10         GGTTCCATGGTGTAATGGTTAGCACTCTGGACTCTGAATCCAGCGATCCGAGTTCAAATCTCGGTGGAACCTCCA
SPRET_EiJ    GGTTCCATGGTGTAATGGTTAGCACTCTGGACTCTGAATCCAGCGATCCGAGTTCAAATCTCGGTGGAACCTCCA
WSB_EiJ      GGTTCCATGGTGTAATGGTTAGCACTCTGGACTCTGAATCCAGCGATCCGAGTTCAAATCTCGGTGGAACCTCCA
129S1_SvIm[illegible]GGTTCCATGGTGTAATGGTTAGCACTCTGGACTCTGAATCCAGCGATCCGAGTTCAAATCTCGGTGGAACCTCCA
DBA_2J       GGTTCCATGGTGTAATGGTTAGCACTCTGGACTCTGAATCCAGCGATCCGAGTTCAAATCTCGGTGGAACCTCCA
C3H_HeJ      GGTTCCATGGTGTAATGGTTAGCACTCTGGACTCTGAATCCAGCGATCCGAGTTCAAATCTCGGTGGAACCTCCA
CBA_J        GGTTCCATGGTGTAATGGTTAGCACTCTGGACTCTGAATCCAGCGATCCGAGTTCAAATCTCGGTGGAACCTCCA
NZO_HlLtJ    GGTTCCATGGTGTAATGGTTAGCACTCTGGACTCTGAATCCAGCGATCCGAGTTCAAATCTCGGTGGAACCTCCA
AKR_J        GGTTCCATGGTGTAATGGTTAGCACTCTGGACTCTGAATCCAGCGATCCGAGTTCAAATCTCGGTGGAACCTCCA
A_J          GGTTCCATGGTGTAATGGTTAGCACTCTGGACTCTGAATCCAGCGATCCGAGTTCAAATCTCGGTGGAACCTCCA
CAST_EiJ     GGTTCCATGGTGTAATGGTTAGCACTCTGGACTCTGAATCCAGCGATCCGAGTTCAAATCTCGGTGGGACCTCCA
NOD_ShiLtJ   GGTTCCATGGTGTAATGGTTAGCACTCTGGACTCTGAATCCAGCGATCCGAGTTCAAATCTCGGTGGAACCTCCA
BALB_cJ      GGTTCCATGGTGTAATGGTTAGCACTCTGGACTCTGAATCCAGCGATCCGAGTTCAAATCTCGGTGGAACCTCCA
LP_J         GGTTCCATGGTGTAATGGTTAGCACTCTGGACTCTGAATCCAGCGATCCGAGTTCAAATCTCGGTGGAACCTCCA
PWK_PhJ      GGTTCCATGGTGTAATGGTTAGCACTCTGGACTCTGAATCCAGCGATCCGAGTTCAAATCTCGGTGGAACCTCCA
FVB_NJ       GGTTCCATGGTGTAATGGTTAGCACTCTGGACTCTGAATCCAGCGATCCGAGTTCAAATCTCGGTGGAACCTCCA
C57BL_6NJ    GGTTCCATGGTGTAATGGTTAGCACTCTGGACTCTGAATCCAGCGATCCGAGTTCAAATCTCGGTGGAACCTCCA
rn6          GGTTCCATGGTGTAATGGTTAGCACTCTGGACTCTGAATCCAGCGATCCGAGTTCAAATCTCGGTGGAACCTCCA
```

Supplementary Figure S9: Sequence alignment of the ortholog set for the tRNA-Gln-CTG-2-1 locus. The variable nucleotide position is highlighted in yellow, and the CAST/EiJ-specific substitution is indicated in red.
